# The Effect of Physical Activity and Repeated Whole-Body Cryotherapy on the Expression of Modulators of the Inflammatory Response in Mononuclear Blood Cells among Young Men

**DOI:** 10.3390/jcm13092724

**Published:** 2024-05-06

**Authors:** Justyna Kusmierczyk, Magdalena Wiecek, Gabriela Wojciak, Mateusz Mardyła, Grzegorz Kreiner, Zbigniew Szygula, Jadwiga Szymura

**Affiliations:** 1 Department of Physiology and Biochemistry, University of Physical Education in Kraków, 31-571 Kraków, Poland; magdalena.wiecek@awf.krakow.pl (M.W.); mateusz.mardyla@awf.krakow.pl (M.M.); 2 Department of Biomechanics and Physical Medicine, Institute of Health Sciences, Faculty of Medicine and Health Sciences, Andrzej Frycz Modrzewski Krakow University, 30-705 Kraków, Poland; gwojciak@365-afm.edu.pl; 3 Department Brain Biochemistry, Maj Institute of Pharmacology, Polish Academy of Sciences, 31-343 Kraków, Poland; kreiner@if-pan.krakow.pl; 4 Department of Sports Medicine and Human Nutrition, University of Physical Education in Kraków, 31-571 Kraków, Poland; zbigniew.szygula@awf.krakow.pl; 5 Department of Clinical Rehabilitation, University of Physical Education in Kraków, 31-571 Kraków, Poland; jadwiga.szymura@awf.krakow.pl

**Keywords:** physical activity and cryotherapy interactions, inflammation modulators, systemic anti-inflammatory effects

## Abstract

**Background:** Series of whole-body cryotherapy (WBC) among healthy and physically active individuals can potentially reduce inflammatory response, although exact mechanisms remain unclear. **Methods:** The impact of whole-body cryotherapy on inflammation modulators among 28 young males, categorized as non-training (NTR, N = 10), non-training with WBC (NTR-WBC, N = 10), and training with WBC (TR-WBC, N = 8), is investigated in this study. Over a period of eight weeks, NTR-WBC and TR-WBC subjects underwent 24 WBC treatments (−130 °C for 3 min, three times a week), examining changes in mRNA expressions of IL-1A, IL-6, IL-10, IFN-G, SIRT1, SIRT3, SOD2, GSS, and ICAM-1. **Results:** The received data indicate an acute inflammatory response to initial WBC (increased IL-1A, IL-6, and SIRT), with a greater effect in NTR-WBC. Subsequent sessions showed enhanced expressions of antioxidative genes in both WBC groups, particularly non-trained, suggesting improved oxidative stress adaptation. A notable decrease in ICAM-1 mRNA post-24 WBC treatments in NTR-WBC signifies a potential systemic anti-inflammatory effect. **Conclusions:** The findings of the study suggest that the combination of regular physical activity with WBC administered three times per week can potentially modulate inflammatory and antioxidant responses. This modulation is evidenced by changes in the expression of genes related to these processes.

## 1. Introduction

Whole-body cryotherapy (WBC) is a technique that entails short exposures to subzero temperatures, typically between −100 °C and −160 °C, designed to elicit the body’s natural response to intense cold. This method is used for its preventive and therapeutic benefits, promoting a spectrum of physiological adaptations that can relieve various health issues, bolster metabolism, facilitate the removal of metabolic by-products, and offer anti-inflammatory, anti-edematous as well as analgesic effects [1]. Additionally, WBC is believed to help restore balance to the immune system, activate hormonal responses, and increase antioxidant protection [2,3].

Evidence from research suggests that WBC treatments can alleviate inflammatory responses and muscle trauma associated with intense physical exertion, aiding faster recovery for both regular exercisers and top-level athletes [4]. Despite the growing body of literature on WBC’s application in sports medicine, studies have largely been centered around the recovery phase, with scant attention to its role during training, competition, or intense training periods [5].

Oxidative stress is a key factor impacting athletic performance, as it is a byproduct of muscle activity that can lead to harmful reactions and the production of reactive oxygen species (ROS). These ROS can impair cell membranes and activate the immune system. It is hypothesized that WBC amplifies antioxidative processes, potentially reducing immune system overactivity [6].

An investigation of the Polish national kayak team, who were subjected to two daily WBC sessions during a ten-day training block, observed an initial decrease in glutathione peroxidase (GPx) activity, which normalized after the tenth day of cryotherapy. Following a subsequent nine-day training period without WBC, GPx levels and the concentration of reactive substances with thiobarbituric acid (TBARS) returned to baseline, suggesting that WBC might bolster the elimination efficiency of TBARS, thus optimizing training effectiveness [7].

Conversely, a single session of WBC administered to professional volleyball players led to a decrease in catalase (CAT) activity and an 8% reduction in superoxide dismutase (SOD) activity compared to the control group [8]. A decline in SOD and GPx activities, along with a drop in TBARS levels, was also observed in professional rowers over a six-day training cycle when compared to training without WBC [9].

The number of administered WBC treatments affects antioxidative enzyme activity. While ten sessions did not significantly change SOD and GPx levels in young healthy men, there was a notable increase in CAT activity and levels of reduced as well as oxidized glutathione (GSH and GSSG, respectively). Extending the treatment to 20 sessions induced a significant rise in SOD activity and a decrease in GSH and GSSG levels below the initial baseline [10]. Such adaptations are beneficial for maintaining optimal prooxidative-antioxidative balance and enhancing the body’s defense against oxidative stress, with a minimum of 20 sessions recommended for restoring normal hematological and immunological parameters [11]. Evidence suggests that the antioxidative response to WBC may vary according to the age of the participants [12]. A single session of WBC can affect the prooxidative-antioxidant balance, as indicated by the glutathione disulfide to glutathione ratio (GSSG/GSH) [13]. Furthermore, the implementation of WBC on an every-other-day schedule has been shown to decrease oxidative stress levels both in young individuals who engage in low levels of physical activity and in the elderly, regardless of their present physical activity levels [13].

The anti-inflammatory and pain-relieving properties of WBC have been the subject of research in both athletic and clinical settings. While changes in immune system markers after WBC sessions are indicated in some studies, others find no significant correlation [14]. However, the consensus is that WBC helps diminish pain, improve mood, and increase quality of life; nonetheless, the need to conduct additional research is highlighted [15].

The effects of WBC on inflammatory response were measured not only in professional athletes but also in healthy individuals and patients with inflammatory diseases [16]. In a study involving young, healthy males, IL-6 levels surged significantly 30 min and 24 h after a single WBC session, more so than after ten days of treatment [10]. A series of 20 WBC sessions was found to increase anti-inflammatory cytokines and decrease pro-inflammatory ones in healthy men [17]. In a separate study among 45 healthy men, varying effects on immune parameters were reported: five WBC sessions increased IL-10 by 30%, but this was reversed two weeks post-treatment. After ten sessions, levels of IL-1α decreased by 17%, while IL-6 and IL-10 rose by 10% and 14%, respectively. After 20 sessions, cytokine concentrations stabilized at similar levels to those after ten sessions, but the decrease in IL-1α persisted even two weeks post-therapy [18]. Among professional rugby players, a slight decrease in ferritin and transferrin levels was observed, possibly validating the anti-inflammatory effect of WBC [19].

Lastly, sirtuins, which play a crucial role in the body’s oxidative balance, may be influenced by cold exposure. It has been shown in studies on animals that low temperatures can alter sirtuins activity [20], particularly increasing the expression of sirtuin 3, a protein involved in thermogenesis and cold adaptation [21]. These findings open up new avenues for research regarding the effects of extremely low temperatures on these proteins in humans, especially within the context of WBC [12].

The aim of the study is to ascertain the impact of both singular and repeated WBC exposures on modulating expression profiles of inflammatory mediators and enzymes that determine redox equilibrium. The research encompasses young adult males, inclusive of both athletic and non-athletic subjects. The primary investigative metrics include a panel of cytokines comprising both pro-inflammatory and anti-inflammatory agents (IL-1α, IL1-RN, IL-6, IL-10, and IFN-gamma), deacetylases associated with aging—longevity-linked sirtuins (SIRT1, SIRT3), critical antioxidative enzymes (GSS, SOD-2), and intercellular adhesion molecules (ICAM-1). These markers are quantitatively assessed in mononuclear blood cells to delineate systemic responses elicited by WBC.

## 2. Materials and Methods

### 2.1. Study Design

The study included healthy, Caucasian males from three groups (18–30 years) who had been training long-distance running for at least 2 years, and non-training subjects who had no contraindications to the application of WBC treatments. Non-training men were randomly assigned to two groups, a control group and one subjected to WBC treatments. A priori power analysis was performed utilizing G*Power 3.1 software (Dusseldorf, Germany) to determine the requisite sample size for the study [22]. Considering the experiment’s structure of three groups and four measurement points, alongside a predefined alpha of 0.05 and a test power of 0.80, it was calculated that a total of 30 subjects (10 per group) would be required to adequately power the study.

After medical qualification, men who met the inclusion criteria underwent 24 WBC treatments, which were performed three times a week (Monday, Wednesday, and Friday) for eight consecutive weeks. The treatments were performed in the early afternoon. The subjects were asked not to change their eating or physical activity habits during the whole study period.

Before and after 1 WBC, following 12 WBC, and after 24 WBC procedures, the gene expressions in peripheral blood mononuclear cells (PBMC) were determined for each person.

### 2.2. Study Participants

Individuals who presented medical reasons that precluded the use of WBC or had undergone WBC in the past six months were not considered for this study. Additional exclusion criteria included cigarette smoking, alcohol misuse, ongoing medication usage, adherence to restrictive or specific diets such as vegetarianism or veganism, supplementation with dietary aids, active participation in athletic activities other than long-distance running, or having less than two years of experience in this discipline.

From the initial pool of candidates, 38 individuals were accepted for the study. Subsequent to participant enrollment, individuals were cataloged in a database and successively numbered. The volunteers without training were randomized via an Excel macro to be allocated to either a control cohort or to the group designated for WBC intervention. However, ten participants withdrew during the course of the project. The final data set was composed of 28 men, divided into the following groups:

(1) TR-WBC, young men with experience in endurance training (average training duration of 3.35 ± 1.83 years);
(2) NTR-WBC, young men without training regimes;
(3) NTR, another cohort of young untrained men, who did not participate in the WBC sessions.

The achieved test power for statistical analysis with a sample size of 28 participants is 0.78.

The study was conducted in accordance with the 1964 Declaration of Helsinki. The methodology of the study was approved by the Bioethical Committee of the Regional Medical Chamber (285/KBL/OIL/2020, 18 December 2020).

### 2.3. Whole-Body Cryotherapy Procedure

The whole-body cryotherapy routine involved an initial 30-s period in an antechamber set to the temperature of −60 °C, followed by three-minute exposure in the primary cryochamber, which was maintained at −130 °C. This environment was achieved by utilizing liquid nitrogen to cool the air. These sessions took place in the KN-1 model cryogenic chamber manufactured by Bamet, based in Wielka Wieś, Poland. The oxygen levels within the cryochamber were consistently maintained between 21–22% and were constantly monitored by two separate oxygen sensors (model EurOx.O2 G/E from Krakow, Poland).

The protocol allowed for the participation of up to four individuals simultaneously. Throughout the duration of the treatment, individuals would walk in a line, circling the chamber, and according to the given auditory cue, they would reverse their direction, all the while maintaining a steady breathing pattern without abrupt changes.

Participants were clad in athletic shorts, knee-high socks, clogs, gloves, ear-covering caps, and surgical masks with gauze over the mouths and noses for the duration of the cryotherapy. Prior to entering the cryochamber, they were required to remove eyewear, watches and other accessories. In order to prevent frostbite, each participant was instructed to thoroughly dry off and to avoid rubbing their skin while undergoing the treatment.

The cryotherapy facility was outfitted with features such as a real-time temperature monitoring system for both chambers, an automated air drying mechanism, sensors for detecting oxygen concentration, and a video surveillance setup to monitor the chamber’s interior. An emergency button and a lever for instant door release from within were also present for safety. Direct visual supervision was possible through thermally insulated glass and communication was maintained with a camera system. All procedures were conducted under the supervision of certified physical therapists.

### 2.4. Assessment of Body Composition

Prior to initiating the sequence of whole-body cryotherapy sessions, each individual’s body height and body mass were recorded. Evaluation of body composition was carried out through electrical bioimpedance analysis (BIA), employing a multifrequency bioelectrical impedance device with eight electrodes operating at frequencies of 5 kHz, 50 kHz, and 250 kHz (Jawon IOI-353 Body Composition Analyzer, from Gyeongsa, Korea). For all participants, body mass index (BMI) was computed. These findings are presented in Table 1.

### 2.5. Venipuncture and Blood Collection

Venous blood was drawn on four separate occasions: prior to the initial whole-body cryotherapy session (pre-1 WBC), 30 min after completing: the 1st session (post-1 WBC), after the 12th session (post-12 WBC), and following the completion of 24th session (post-24 WBC), utilizing a vacuum extraction system (produced by Becton Dickinson, located in Franklin Lakes, NJ, USA). The procedure required participants to sit still for five minutes before blood was drawn.

For the purpose of analyzing mRNA expression, blood was drawn into 4 mL Vacutainer BD CPT™ (Becton Dickinson, Franklin Lakes, NJ, USA) tubes, which contain sodium citrate and FICOLL™. This combination allows for a streamlined and standardized procedure to separate PBMCs—lymphocytes and monocytes—from whole blood. The blood samples were then centrifuged for 15 min at a relative centrifugal force (RCF) of 3000× *g* using the MPW-351R centrifuge at 21 °C (from MPW Med. Instruments, based in Warsaw, Poland). PBMC was preserved at a temperature of −80 ± 5 °C until the time of analysis, using the ZLN-UT 300 PREM freezer from POL-EKO-APARATURA in Wodzisław Śląski, Poland.

### 2.6. mRNA Expression Analysis

The expression of 12 mRNA coding for various proteins—interleukin 1 alpha (IL-1A), interleukin 1 receptor antagonist (IL-1RN), interleukin 6 (IL-6), interleukin 10 (IL-10), tumor necrosis factor alfa (TNF A), interferon gamma (IFN-G), sirtuin 1 (SIRT1), sirtuin 3 (SIRT3), glutathione synthetase (GSS), superoxide dismutase 2 (SOD2), intercellular adhesion molecule 1 (ICAM1), and beta-2-microglobulin (B2M)—was selected for analysis based on synthesis of the research results [6,16,17,18,19,20].

#### 2.6.1. mRNA Isolation and Quality Control of RNA

RNA was isolated from PBMCs using a commercial column-based extraction kit according to the manufacturer’s protocol (Total RNA Mini, A&A Biotechnology, Gdańsk, Poland). In brief, PBMCs were resuspended on ice post-thaw and centrifuged at 3000× *g* for five minutes at 4 °C. Subsequently, 200 µL of PBMCs was transferred to a new tube, and 750 µL of Fenozol Plus was added. The RNA was then eluted with 50 µL of ultrapure water, incubated for three minutes, and centrifuged at 15,000× *g* for one minute at room temperature. The RNA was stored at −80 ± 5 °C. Purity and concentration were assessed via spectrophotometry at 260, 280, and 230 nm wavelengths, with ratios of A260:A230 > 1.7 and A260:A280 > 2.0 indicating a high level of purity.

#### 2.6.2. Reverse Transcription

The isolated RNA underwent reverse transcription using a high-capacity cDNA synthesis kit (TaqMan^®^ High-Capacity cDNA Reverse Transcription Kit, Thermo Fisher Scientific, Waltham, MA, USA). The cDNA synthesis protocol entailed incubation at 16 °C for 30 min, 42 °C for 30 min, and then 85 °C for five minutes. The cycler was then set to 4 °C to terminate the reaction. The resultant cDNA was stored at −20 ± 2 °C until further analysis.

#### 2.6.3. Real-Time Quantitative PCR

For real-time qPCR, the Applied Biosystems TaqMan^®^ Fast Advanced Master Mix was used (Cat. No. 444557, Thermo Fisher Scientific, Waltham, MA, USA), alongside specific TaqMan^®^ mRNA Assays (Cat. No. 4331182, Thermo Fisher Scientific, Waltham, MA, USA) for each gene: IL-1A (Hs00174092_m1), IL-1RN (Hs00893626_m1), IL-6 (Hs00174131_m1), IL-10 (Hs00961622_m1), TNF-A (Hs00174128_m1), IFN-G (Hs00989291_m1), SIRT1 (Hs01009006_m1), SIRT3 (Hs00953477_m1), GSS (Hs00609286_m1), SOD2 (Hs00167309_m1), ICAM1 (Hs00164932_m1), and B2M (Hs00187842_m1). Each sample was assayed in duplicate. The PCR protocol began with a cycle at 95 °C for ten minutes, followed by 40 cycles of 95 °C for 15 s and 60 °C for one minute. Fluorescence signals were collected at the end of each cycle to determine cycle threshold (Ct) values. The fold changes in mRNA expression were calculated using the relative quantification (RQ) method, where ΔCt sample = Ct_target − Ct_HK, ΔΔCt = ΔCt sample − average ΔCt control group, and RQ = 2^−ΔΔCt^ [23]. Fold-change values above one suggest up-regulated gene expression, while values below one indicate down-regulation.

### 2.7. Statistical Analysis

All statistical analyses were conducted using the STATISTICA 13.3 software package (StatSoft, Inc., Tulsa, OK, USA). To evaluate the distribution patterns of the variables under study, the Shapiro–Wilk test was utilized to assess normality, and Levene’s test was applied to confirm the homogeneity of variances. The assessment of differences between groups for individual measurements (age, body composition, medical qualification) employed appropriate statistical tests based on the data distribution of one-way ANOVA, Student’s *t*-test for variables with a normal distribution and the non-parametric Kruskal–Wallis as well as Mann–Whitney U tests for those that were not normally distributed. The analysis of changes in expression within the experimental groups was conducted using Student’s *t*-test, which included comparisons between post-treatment and baseline values.

The *p*-value ≤ 0.05 was established as the threshold for statistical significance in the observed differences.

## 3. Results

### 3.1. Characteristics of the Study Participants

#### 3.1.1. Somatic Characteristics

Significant differences were observed in the non-training (NTR) group compared to the WBC groups (NTR-WBC and TR-WBC) in terms of percentage of body fat (PBF) and mass body fat (MBF), with existing statistical significance (PBF: *p* = 0.03; MBF: *p* = 0.04). These variations are detailed in Table 1.

#### 3.1.2. Hematological and Biochemical Indices of the Participants

Significant differences were found among the groups in various hematological and biochemical indices. The TR-WBC group exhibited lower hemoglobin concentration compared to others (*p* = 0.05), while the NTR-WBC group showed a higher hematocrit value and a lower platelet count than the other groups, with statistical significance (hematocrit: *p* = 0.03; platelet count: *p* = 0.04), as detailed in Table 2. 

### 3.2. Selected Gene Expressions

In the study, significant changes in mRNA expression were observed post-WBC treatment in the TR-WBC (training) group and the NTR-WBC (non-training) group. These changes are summarized in Table 3.

#### Comparisons of Changes between the Number of WBC Sessions and Baseline Values

Changes following the first WBC session compared to pre-treatment values included significant increases in IL-1A (*p* = 0.001), IL-6 (*p* < 0.001), and SIRT1 mRNA levels (*p* = 0.012) in the TR-WBC group. The NTR-WBC group showed increases in IL-1A (*p* = 0.004), IL-6 (0.001), and SIRT-1 mRNA levels (*p* = 0.019) (Table 3).

After 12 WBC sessions, both TR-WBC and NTR-WBC groups exhibited further increases in SIRT-1 (TR-WBC: *p* = 0.003, NTR-WBC: *p* = 0.007) and IL-6 (TR-WBC: *p* = 0.018, NTR-WBC: *p* <0.001). In the TR-WBC group, there was also a decrease in ICAM-1 mRNA levels (*p* < 0.001). In the NTR-WBC group, a significant increase in SIRT3 (*p* = 0.03) and decrease in ICAM-1 mRNA levels (*p* < 0.001) were noted. 

By the 24th WBC session, in the NTR-WBC group, IL-6 (*p* = 0.005), SIRT1 (*p* = 0.022), SIR3 (*p* = 0.01), SOD2 (*p* = 0.009), and GSS (*p* = 0.046) mRNA levels were increased while ICAM1 values experienced a decrease (*p* = 0.05).

### 3.3. Comparison of Baseline Values in Non-Training and Training Groups

Comparing the resting values of the two non-training groups with those of the training group using the Student’s *t*-test, down-regulation was observed in mRNA expression for ICAM-1 (*t* = 3.85, *p* = 0.00067) and up-regulation in SIRT1 expression (*t* = −2.31, *p* = 0.03). No changes were noted for other transcripts.

## 4. Discussion

In the present investigation, a unique investigation was undertaken regarding the influence of recurrent whole-body cryotherapy sessions on gene transcription profiles within mononuclear blood cells. The aim of the study was to evaluate the effects of repeated whole-body cryotherapy sessions on the expression of genes related to inflammation and the antioxidant system in young males, both among trained athletes and untrained individuals. The foundational reasoning for focusing on mRNA in research is fundamentally because it acts as a nuanced barometer for the cellular environment, responding to stimuli [24]. By comparison, protein levels change more gradually, and thus the dynamic nature of mRNA offers an early detection system for transcriptional changes that precede protein expression [25]. Investigating mRNA not only provides information on the existing protein landscape but also opens a gateway to understanding the intricate regulatory networks that govern gene expression [26]. It also enables the identification of alternative splicing events, therefore contributing to proteomic heterogeneity. This knowledge is crucial, as it aids the identification of potential targets for therapy and enhances comprehension of the underlying mechanisms influenced by WBC [27].

In the present observations of post-WBC, there was an augmentation in the mRNA expression of GSS, SOD2, SIRT1, and SIRT3. This concurs with the findings of dos Santos Silva et al. on post-cryotherapy elevations in glutathione [28]. Moreover, Wojciak et al. reported altered SOD, Sirt1, and Sirt3 protein activities, reflecting cellular adaptation to oxidative stress, which was also noted in the current trial [19]. Delayed changes in mRNA levels for GSS and SOD2 may suggest a transcriptional response, potentially leading to these protein-level changes. Such a temporally delayed transcriptional response [29] may be emblematic of an enduring cellular accommodation to repetitive cytotherapeutic stimuli, serving to modulate antioxidant capacities, especially in subjects with infrequent exposure to physical exertion [30].

Additionally, in the research conducted by Qu et al., a physiological framework was established, demonstrating a downtrend in muscle soreness and inflammatory biomarkers following WBC sessions [31]. These clinical observations are congruent with the molecular data obtained in the current study, which illustrate an upsurge in the mRNA levels of genes associated with antioxidative function and mitochondrial integrity, possibly reflecting an augmented recovery process and attenuation of inflammatory responses. Notably, the reduction of mRNA expression in ICAM-1 observed post-WBC is in harmony with the findings of Peyronnel et al., indicative of a systemic anti-inflammatory effect [32]. Analysis of expression using a greater number of time points could contribute to understanding the mechanisms of WBC action [33].

The observed increase in the expression of antioxidative genes within the non-trained group offers notable results, indicating potential enhancement of the body’s defense mechanisms against oxidative stress due to WBC interventions [34]. Such an effect can promote this kind of therapy in varied patient populations, with chronic inflammatory conditions or metabolic disorders, where oxidative stress is a contributing factor [35,36]. Further exploration into these physiological and clinical influences could progress the understanding concerning the therapeutic potential of WBC and its role in preventive medicine.

A single session of WBC elicited upregulation in IL-1A mRNA abundance, accompanied by elevation of IL-6 transcript levels. Such a finding is consistent with expectations, given the known rapid mobilization of IL-1A during the plasma in the wake of stimulatory exposure [37]. Normalization of IL-1A levels was observed subsequent to a dozen treatment sessions. The determination of optimal temporal markers for transcriptomic changes poses a methodological challenge [38]. Moreover, both physical activity and WBC increased the expression of IL6 mRNA, and this effect could be observed during the whole study, which may indicate the influence of WBC on the modulation of inflammatory pathways [39]. The noted anti-inflammatory properties of WBC indicate its potential implementation as an adjunct therapeutic intervention in conditions marked by chronic low-grade inflammation, such as metabolic syndrome [40] atherosclerosis [41], and multiple sclerosis [42], but also cardiovascular diseases [43] and mental disorders [44]. 

The strength of the following study lies in determining the effect of exercise and WBC on modulators of inflammation and oxidative stress at the level of gene expression. This fills a gap in the current understanding of these connections and allows us to demonstrate the benefits of combining cryotherapy with exercise training to maximize health and performance. Building upon this investigation, future research should be carried out to examine the molecular mechanisms underlying changes in gene expression induced by WBC. This includes assessing modifications of antioxidant levels within muscle tissue, with specific focus on mitochondrial function, to fully understand the impact of WBC on oxidative balance and myocyte metabolism [45]. Furthermore, longitudinal monitoring of physiological and biochemical markers is essential to confirm the hypothesized benefits of WBC in enhancing post-exercise recovery [46]. A comprehensive analysis of WBC effects on muscle hypertrophy and anabolic processes is warranted [47]. Exploring novel strategies that incorporate WBC prior to exercise could reveal its timing-dependent efficacy in promoting muscle repair and growth [48]. Such strategies may also offer insights into how WBC could modulate mTOR kinase pathways, potentially priming the musculoskeletal system against exercise-induced stress [49].

The limitation of the study, was primarily the small number of volunteers and the restricted profile of the analyzed mRNA. A smaller sample size introduces greater susceptibility to random variations and potentially limits the representation of a broader population. Therefore, caution must be taken when generalizing these findings to larger and more diverse demographic groups. Nevertheless, every effort has been made to minimize the influence of extraneous factors on the results. This includes controlling the diet and ensuring the inclusion of individuals with similar physical training backgrounds. To enhance the robustness and applicability of the research, it is advisable to extend the scope to a larger and more varied cohort of participants, particularly long-distance runners, in order to include a fourth group (TR without WBC). Moreover, the study duration and follow-up period should be considered; a lengthened timeline or a more extensive follow-up could yield more insights into the longer-term effects of the intervention. The absence of long-term follow-up data curtails the ability to determine permanence of the results beyond the span of the immediate study period.

In summary, while protein analysis remains pivotal in life sciences, exploring mRNA levels offers new understandings regarding gene regulation and cellular responses. This approach unveils novel aspects of cellular function not evident through protein-level analysis alone. The interplay between exercise, cytokine levels, oxidative stress, and radical formation, and how WBC influences these relationships, warrants further investigation. Integrating findings from other studies, and those from this investigation, allows us to highlight the complex influence of WBC on cytokine modulation and immune dynamics, suggesting its potential as a therapeutic modality in sports and broader health contexts [39,50,51,52].

## 5. Conclusions

The present research is at the forefront of exploring modulation of gene expression in the mononuclear blood cells of young men in response to repeated sessions of whole-body cryotherapy, with focus on mRNA analysis. The findings suggest the following:

(1) Repeated exposure to WBC augments the expression of genes associated with antioxidative defense;
(2) The upregulation of IL-1A and IL-6 mRNA abundance following WBC sessions possibly causes an acute inflammatory response, which normalizes with repeated treatments, indicating potential modulation of inflammatory pathways by WBC;
(3) An adaptive response to WBC is comparable to the benefits derived from habitual physical exercise;
(4) Despite limitations, the study underscores the potential of WBC as a therapeutic modality in sports and broader health contexts.

By combining the evaluation of both mRNA and protein levels, this study contributes fresh perspectives to the understanding of cellular functionality and the potential health benefits of WBC. The confluence of data propels knowledge in molecular biology and the biomedical field forward. However, to fully unravel the molecular pathways and mechanisms at play, additional in-depth investigations are warranted.

## Figures and Tables

**Table 1:** Somatic characteristic of participants.

Variable	NTR	NTR-WBC	TR-WBC	*p*-Value
Age [years]	22.40 ± 2.84	21.80 ± 1.40	22.63 ± 1.77	0.69
BH [cm]	181.56 ± 9.61	181.05 ± 7.51	182.25 ± 6.23	0.95
BM [kg]	83.49 ± 7.14	77.67 ± 6.96	78.35 ± 11.55	0.28
PBF [%]	22.25 ± 5.68	16.09 ± 5.17 *	15.60 ± 6.10 *	0.03
BF [kg]	18.66 ± 5.14	12.51 ± 4.23 *	12.35 ± 6.25 *	0.02
LBM [kg]	64.83 ± 6.66	65.16 ± 6.69	66.00 ± 10.27	0.95
TBW [%]	46.67 ± 4.80	46.90 ± 4.83	47.53 ± 7.40	0.95
SLM [kg]	60.00 ± 6.35	60.59 ± 6.37	61.43 ± 9.69	0.92
BMI [kg/m^2^]	25.47 ± 2.95	23.74 ± 2.20	23.54 ± 2.80	0.24

Values are means ± SD; BM: body mass, BH: body height, PBF: percentage of body fat, BF: body fat, LBM: lean body mass, TBW: total body water: SLM: soft lean mass, BMI: body mass index (BMI = BM (kg)/BH (m)^2^). * *p* ≤ 0.05 vs. NTR. Statistically significant differences were measured using ANOVA.

**Table 2:** Hematological and biochemical indices of participants.

Variable	NTR	NTR-WBC	TR-WBC	*p*-Value
RBC (10^6^/µL)	5.12 ± 0.20	5.21 ± 0.28	4.96 ± 0.33	0.29
HGB (g/dL)	15.66 ± 0.94	15.97 ± 0.70	14.68 ± 0.93 *$	0.01
HCT (%)	44 ± 2.03	46.27 ± 2.14 *#	43.23 ± 2.57	0.02
PLT (1000/µL)	243.2 (177–305)	212.0 (200–226) *#	241.5 (226–286)	0.04
LEUC (1000/µL)	7.69 (4.49–19.72)	7.09 (5.23–8)	5.46 (4.9–7.13)	0.52
NEUT (%)	52.27 ± 12.89	49.70 ± 14.03	49.56 ± 13.11	0.86
LYMPH (%)	34.49 ± 10.74	36.91 ± 13.18	35.89 ± 10.61	0.87
MONO (%)	9.10 ± 1.79	9.47 ± 1.53	9.85 ± 2.66	0.58
EOS (%)	3.52 (0.10–7.80)	3.2 (2.2–4.0)	3.3 (2.2–5.1)	0.68
BASO (%)	0.62 ± 0.34	0.72 ± 0.31	0.73 ± 0.47	0.72
Glucose (mmol/L)	5.04 (4.46–5.57)	4.82 (4.67–4.94)	5.02 (4.73–5.16)	0.35
CHOL (mmol/L)	4.29 ± 0.89	4.19 ± 1.04	4.24 ± 1.03	0.93
HDL-C (mmol/L)	1.34 ± 0.19	1.47 ± 0.45	1.61 ± 0.28	0.88
LDL-C (mmol/L)	2.54 (1.57–3.93)	2.34 (1.94–2.57)	1.82 (1.66–2.71)	0.93
TG (mmol/L)	0.90 ± 0.27	0.87 ± 0.25	0.78 ± 0.24	0.45
SBP (mmHg)	124.00 ± 5.16	120.50 ± 13.83	120.50 ± 16.24	0.43
DBP (mmHg)	70.00 ± 6.67	70.50 ± 7.98	74.50 ± 9.56	0.67

Arithmetic mean ± SD or median and quartiles (Q1–Q3); * statistically significant differences (*p* ≤ 0.05) vs. NTR, # statistically significant differences (*p* ≤ 0.05) vs. TR-WBC, $ statistically significant differences (*p* ≤ 0.05) vs. NTR-WBC, RBC—red blood cells, HGB—hemoglobin, HCT—hematocrit, PLT—platelets, LEUC—white cells, NEUT—neutrophils, LYMPH—lymphocytes, MONO—monocytes, EOS—eosinophils, BASO—basophils, CHOL—total cholesterol, HDL-C—high-density lipoproteins cholesterol, LDL-C—low-density lipoproteins cholesterol, TG—triglycerides, SBP—systolic blood pressure, DBP—diastolic blood pressure.

**Table 3:** Comparing expression of selected mRNAs in groups and changes under the influence of whole-body cryotherapy.

Variable	Group	Before 1 WBC	After 1 WBC	After 12 WBC	After 24 WBC	Student’s *t*-Test (*p*-Value)		
		Mean ± SD (Relative Fold Change)				After 1 vs. Before	After 12 vs. Before	After 24 vs. Before
IL-1A	NTR	1.37 ± 1.31	1.64 ± 1.55	1.46 ± 1.44	1.5 ± 1.34	0.68	0.87	0.82
	NTR-WBC	1.00 ± 0.55	3.78 ± 2.63	1.61 ± 1.4	1.26 ± 0.83	0.00	0.22	0.42
	TR-WBC	1.42 ± 0.68	3.25 ± 0.99	1.19 ± 1.03	1.06 ± 0.63	0.00	0.6	0.29
IL-6	NTR	1.3 ± 0.55	1.24 ± 0.48	1.21 ± 0.60	1.23 ± 0.41	0.81	0.69	0.76
	NTR-WBC	1.26 ± 0.46	2.16 ± 0.61	2.76 ± 0.76	2.03 ± 0.62	0.00	0.00	0.01
	TR-WBC	1.84 ± 0.38	3.38 ± 0.74	2.49 ± 0.59	1.77 ± 0.64	0.00	0.02	0.82
IL-10	NTR	1.41 ± 1.42	1.54 ± 1.55	1.59 ± 0.97	1.43 ± 1.4	0.85	0.75	0.97
	NTR-WBC	1.02 ± 0.59	1.67 ± 0.70	1.57 ± 1.12	1.31 ± 0.78	0.08	0.18	0.35
	TR-WBC	1.49 ± 0.83	1.63 ± 1.05	1.65 ± 0.82	1.09 ± 0.66	0.76	0.7	0.31
IFNG	NTR	1.39 ± 1.27	1.18 ± 1.07	1.24 ± 1.74	1.17 ± 0.72	0.68	0.83	0.63
	NTR-WBC	1.22 ± 1.27	1.54 ± 0.62	1.88 ± 1.35	1.38 ± 0.5	0.47	0.27	0.72
	TR-WBC	1.05 ± 1.02	2.03 ± 2.39	0.87 ± 0.52	1.29 ± 1.47	0.31	0.66	0.71
SIRT1	NTR	1.19 ± 0.89	1.23 ± 0.95	1.14 ± 0.59	1.22 ± 0.95	0.92	0.88	0.94
	NTR-WBC	0.91 ± 0.38	1.55 ± 0.69	1.83 ± 0.88	1.67 ± 0.87	0.02	0.01	0.02
	TR-WBC	1.82 ± 0.27	2.48 ± 0.6	2.55 ± 0.51	2.05 ± 1.03	0.01	0.00	0.65
SIRT3	NTR	0.97 ± 0.34	1.41 ± 1	1.3 ± 1.08	1.53 ± 1.92	0.2	0.37	0.37
	NTR-WBC	1.09 ± 0.46	1.78 ± 1.6	1.79 ± 0.63	2.79 ± 1.75	0.2	0.00	0.01
	TR-WBC	1.66 ± 1.05	2.45 ± 0.85	2.41 ± 1.74	1.87 ± 1.45	0.12	0.32	0.74
IL-1RN	NTR	1.26 ± 0.79	1.59 ± 1.05	1.27 ± 1.12	1.39 ± 1.08	0.44	0.98	0.76
	NTR-WBC	1.23 ± 0.66	1.44 ± 0.47	1.18 ± 0.37	1.09 ± 0.44	0.41	0.87	0.59
	TR-WBC	1.22 ± 0.38	1.39 ± 0.77	0.99 ± 0.43	0.91 ± 0.36	0.57	0.28	0.12
GSS	NTR	1.18 ± 0.61	0.95 ± 0.56	1.16 ± 0.54	1.21 ± 1.07	0.4	0.96	0.93
	NTR-WBC	1.23 ± 0.78	1.7 ± 0.62	1.79 ± 1.31	2.29 ± 1.35	0.15	0.25	0.05
	TR-WBC	1.52 ± 0.74	2.41 ± 1.31	2.39 ± 1.32	1.75 ± 0.48	0.12	0.13	0.48
SOD2	NTR	1.36 ± 0.74	0.89 ± 0.27	1 ± 0.48	0.98 ± 0.39	0.24	0.38	0.38
	NTR-WBC	1.06 ± 0.36	1.31 ± 0.66	1.37 ± 0.65	1.54 ± 0.37	0.44	0.33	0.01
	TR-WBC	1.49 ± 1.00	1.58 ± 1.33	1.55 ± 0.93	1.34 ± 0.59	0.88	0.9	0.71
ICAM1	NTR	1.12 ± 0.6	1.35 ± 0.89	0.91 ± 0.2	1.12 ± 0.48	0.5	0.32	0.99
	NTR-WBC	1.29 ± 0.67	1.37 ± 0.83	0.41 ± 0.15	0.39 ± 0.30	0.82	0.00	0.00
	TR-WBC	0.33 ± 0.21	0.45 ± 0.19	0.7 ± 0.17	0.5 ± 0.34	0.25	0.00	0.23

## Data Availability

The datasets used and/or analyzed during the current study are available from the corresponding author upon reasonable request.
